# Photon-counting CT of midventricular takotsubo cardiomyopathy

**DOI:** 10.1016/j.radcr.2026.06.018

**Published:** 2026-07-06

**Authors:** Ismaiel Chikh Bakri, André Euler

**Affiliations:** Department of Radiology, Kantonsspital Baden, affiliated Hospital for Research and Teaching of the Faculty of Medicine of the University of Zurich, Im Ergel 1, 5404 Baden, Switzerland

**Keywords:** Takotsubo cardiomyopathy, Midventricular variant, Photon-counting CT, Cardiac CT, Cine CT

## Abstract

Midventricular Takotsubo cardiomyopathy is an atypical variant of stress-induced cardiomyopathy characterized by circumferential midventricular hypokinesia with basal and apical sparing. We present the case of a 63-year-old woman presenting with acute chest pain and elevated cardiac troponin I following emotional stress. While echocardiography initially suggested apical involvement, Photon-Counting Detector Coronary CT Angiography (PCD-CCTA) provided a comprehensive “1-stop-shop'' assessment. The high-resolution photon-counting acquisition excluded obstructive coronary artery disease, while multiphase cine reconstructions definitively characterized the midventricular wall-motion abnormalities. Diagnosis was confirmed by the complete resolution of wall-motion abnormalities on follow-up cardiac MRI. This case highlights the capability of emerging photon-counting CT technology to simultaneously assess coronary anatomy and ventricular function, facilitating the rapid diagnosis of atypical cardiomyopathy variants.

## Introduction

Takotsubo cardiomyopathy is an acute syndrome characterized by transient left ventricular systolic dysfunction that can clinically resemble acute coronary syndrome [1,2]. While the apical form is most common, midventricular variants occur less frequently and may be underrecognized [1,2]. Unlike the apical pattern, which classically demonstrates apical systolic dysfunction with basal sparing or hypercontractility, the midventricular variant is characterized by dysfunction of the mid-left ventricular segments with relative preservation of both basal and apical contraction. Photon-counting detector CT is an emerging technology that may enable improved cardiac imaging through enhanced spatial resolution and reduced electronic noise [3,4]. In cardiac applications, this may support coronary lumen assessment, spectral reconstructions, and multiphase functional analysis within the same examination [[3], [4], [5]]. We present a case of midventricular Takotsubo cardiomyopathy evaluated with photon-counting detector coronary CT angiography (PCD-CCTA), including functional cine analysis, polar map correlation, and follow-up cardiac MRI demonstrating complete recovery.

## Case presentation

A 63-year-old woman presented to the emergency department with acute retrosternal pain after emotional stress. High-sensitivity cardiac troponin I was elevated at 617 ng/L, decreasing to 545 ng/L after 2 hours. Electrocardiography showed no T-wave changes. An initial point-of-care echocardiogram performed in the emergency department suggested possible apical hypokinesia with moderately reduced left ventricular ejection fraction. A repeat transthoracic echocardiogram performed at the cardiology department clarified the pattern as predominantly midventricular hypokinesia (Movie 1).

Photon-counting detector coronary CT angiography (PCD-CCTA) was performed to exclude obstructive coronary artery disease. The examination was performed on a first-generation dual-source photon-counting CT scanner (NAEOTOM Alpha, Siemens Healthcare GmbH, Forchheim, Germany) using retrospective ECG-gating with ECG-based dose modulation. The average heart rate during acquisition was 71 beats/min. Before image acquisition, the patient received 2 doses of sublingual nitroglycerin for coronary vasodilation. No beta-blocker was administered. Coronary arteries were nonobstructive (Fig. 1). Multiphase functional analysis demonstrated a left ventricular ejection fraction of 48%, with akinesia predominantly involving the anterior midventricular wall and mild residual akinesia of the inferior midventricular wall, while basal and apical segments contracted normally. The combined anterior and inferior involvement accounted for the circumferential midventricular hypokinesia pattern on the polar map (Fig. 2). Cine reconstructions (Movie 2) clearly depicted these wall-motion abnormalities. A representative 2-chamber and 3-chamber reconstruction were added to show the basal and apical sparing and the midventricular wall-motion abnormality in a static format (Fig. 3). The patient received conservative management with temporary escalation of heart failure therapy, which was maintained until follow-up cardiac MRI. Follow-up cardiovascular magnetic resonance imaging performed 1 month later (Movie 3) demonstrated complete resolution of wall-motion abnormalities, confirming the transient nature of the disease [6].

**Fig. 1:**
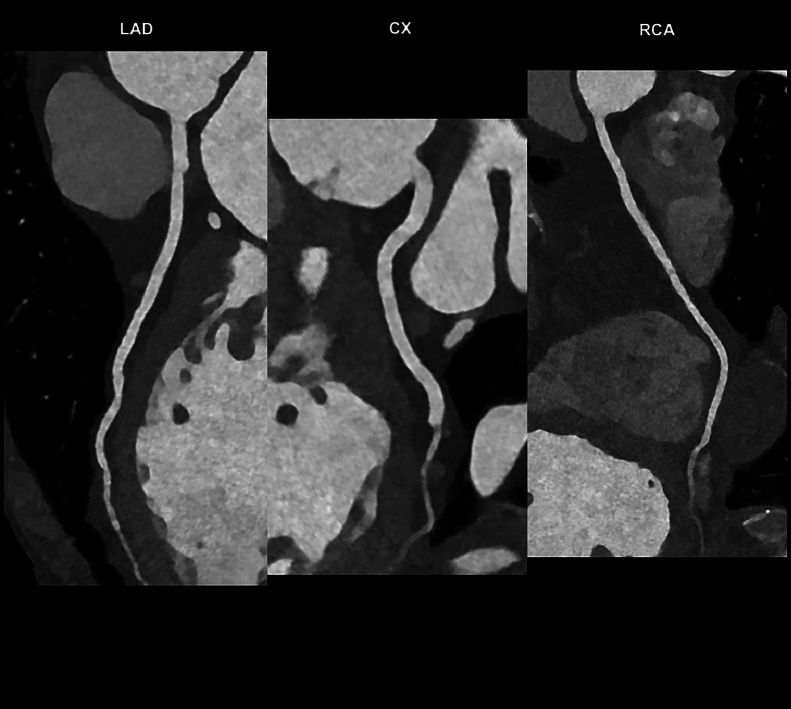
(Curved multiplanar coronary reformations) demonstrates patency of the left anterior descending, left circumflex, and right coronary arteries without obstructive stenosis.

**Fig. 2:**
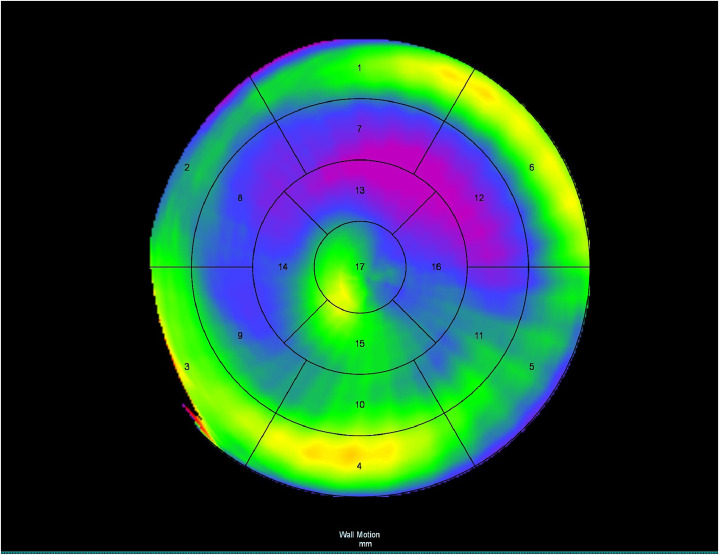
(Polar map of left ventricular wall motion) demonstrates circumferential midventricular hypokinesia consistent with the midventricular Takotsubo pattern.

**Fig. 3:**
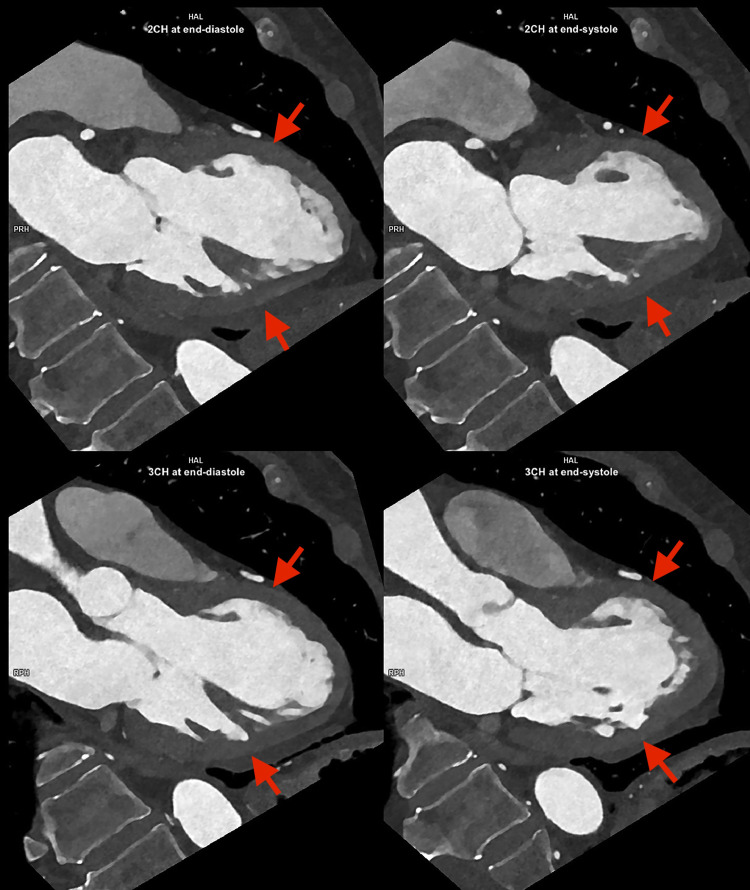
(Two-chamber and 3-chamber PCD-CCTA reconstructions) illustrates the midventricular wall-motion abnormality with preserved basal and apical contraction; arrows indicate the affected midventricular segments, providing a static CT correlate to the cine reconstruction.

## Discussion

Takotsubo cardiomyopathy is a transient cardiomyopathy often triggered by emotional or physical stress and frequently presents with acute chest pain and biomarker elevation, mimicking acute coronary syndrome [1]. Diagnostic evaluation typically requires exclusion of obstructive coronary artery disease and confirmation of transient regional wall-motion abnormalities extending beyond a single epicardial coronary distribution [2].

Several morphologic variants have been described, including apical, midventricular, basal (reverse), and focal patterns [2]. The midventricular form of Takotsubo cardiomyopathy accounts for approximately 10%-15% of cases [1], less frequent than the apical type but more common than the focal variant [7]. Compared with the apical form, the midventricular variant may be less immediately recognizable on limited echocardiographic windows because the apex can contract normally and the abnormality may be confined to the mid-left ventricular ring. This variant can present with balanced midventricular involvement and dynamic recovery, with imaging appearance varying depending on the timing of acquisition. Clinically, it often manifests with chest pain and modest troponin elevation, resembling acute coronary syndrome [1] but typically without obstructive lesions on angiography. The underlying mechanism is thought to involve transient catecholamine-mediated myocardial stunning with regional sympathetic overactivity [2], which may explain the characteristic circumferential midventricular dysfunction.

In this case, PCD-CCTA provided a comprehensive assessment in a single noninvasive examination. First, it excluded obstructive coronary artery disease. Second, multiphase cine reconstructions and functional analysis demonstrated a midventricular pattern of akinesia with preserved basal and apical contraction, consistent with midventricular Takotsubo cardiomyopathy. The polar map offered an intuitive visualization of the circumferential midventricular involvement. Of note, circumferential midventricular dysfunction refers to the involvement of opposing midventricular segments on cine CT and polar map analysis, in contrast to a focal single-segment abnormality.

Photon-counting detector CT may provide advantages for cardiac imaging through direct photon detection, reduced electronic noise, and improved spatial resolution compared with conventional energy-integrating detector CT [3,4]. In coronary CT angiography, these features have been associated with improved objective and subjective coronary image quality and high diagnostic accuracy for coronary artery disease [4,5]. In the present case, the relevant advantages were the combination of high-resolution coronary lumen assessment, retrospective multiphase cine reconstructions for left ventricular function, and polar map visualization in a single acquisition. Compared to traditional energy-integrating CT, PCD-CT potentially increases diagnostic certainty due to reduced image noise and improved spatial resolution [[3], [4], [5]]. Potential limitations of photon-counting CT include limited availability, higher equipment cost, evolving cardiac imaging protocols, reconstruction complexity, and persistent susceptibility to cardiac motion or calcification-related artifacts. The advantages of PCD-CT can support robust functional evaluation and depiction of regional wall-motion abnormalities. Importantly, follow-up cardiac MRI confirmed complete recovery, strengthening the diagnosis and illustrating the reversible nature of Takotsubo cardiomyopathy.

## Conclusion

Photon-counting detector coronary CT angiography can enable integrated evaluation of coronary anatomy and ventricular function at high resolution and reduced image noise and may support the timely diagnosis of atypical Takotsubo variants such as the midventricular form.

## Patient consent

Written informed consent for publication of this case report and accompanying images/videos was obtained from the patient.

## Appendix Supplementary materials

(Transthoracic echocardiography) demonstrates predominantly midventricular hypokinesia. Image, video 1 dummy alt text

(PCD-CCTA multiphase cine reconstruction) shows akinesia predominantly involving the anterior midventricular wall with mild residual akinesia of the inferior midventricular wall, with preserved basal and apical contraction; functional analysis measured a left ventricular ejection fraction of 48%. Image, video 2 dummy alt text

(Follow-up cardiac MRI at 1 month) confirms complete resolution of wall-motion abnormalities. Image, video 3 dummy alt text

## Footnotes

Supplementary material associated with this article can be found, in the online version, at doi:10.1016/j.radcr.2026.06.018.
